# Supplementary material for: The causal relationship between the human gut microbiota and pyogenic arthritis: a Mendelian randomization study
Source: Front Cell Infect Microbiol. 2024 Nov 26;14:1452480. doi: 10.3389/fcimb.2024.1452480 (PMC11629706; doi:10.3389/fcimb.2024.1452480)
Supplement: Supplementary file 1 [file DataSheet1.zip › mendelian test/ebi-a-GCST90027587.csv_forest.pdf]

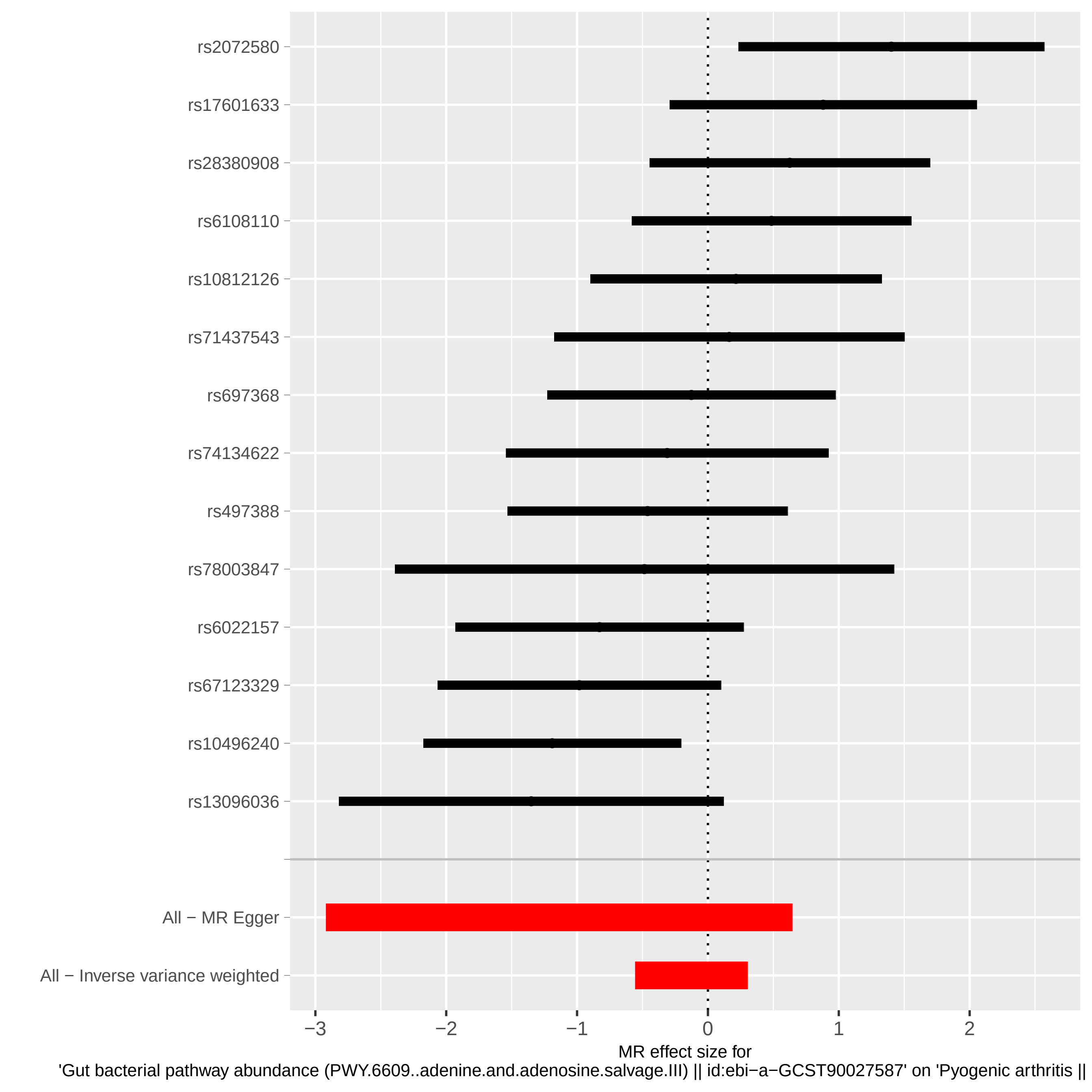
rs2072580
rs17601633
rs28380908
rs6108110
rs10812126
rs71437543
rs697368
rs74134622
rs497388
rs78003847
rs6022157
rs67123329
rs10496240
rs13096036
All − MR Egger
All − Inverse variance weighted
−3
−2
−1
0
1
2
MR effect size for
'Gut bacterial pathway abundance (PWY.6609..adenine.and.adenosine.salvage.III) || id:ebi−a−GCST90027587' on 'Pyogenic arthritis ||
